# Linked avian influenza epidemiological and genomic data in EMPRES-i for epidemic intelligence (2012–2021)

**DOI:** 10.1016/j.dib.2025.111410

**Published:** 2025-02-25

**Authors:** Nejat Arınık, Roberto Interdonato, Mathieu Roche, Maguelonne Teisseire

**Affiliations:** a CRIL UMR 8188, Université d’Artois, Lens, F-62307, France; b INRAE, F-34398 Montpellier, France; c CIRAD, F-34398 Montpellier, France; d TETIS, Univ. Montpellier, AgroParisTech, CIRAD, CNRS, INRAE, Montpellier 34090, France

**Keywords:** Avian influenza, Epidemiology, Data linkage, Transmission dynamics, Network inference

## Abstract

Due to its highly contagious nature, Avian Influenza (AI) is considered an animal health emergency affecting commercial sector and wild bird populations. Several genome sequencing databases have been created to help researchers understand how AI viruses evolve, spread, and cause disease. However, for a global epidemic monitoring approach, they need to be combined to public health surveillance systems, the well-one being EMPRES-i from the World Organisation for Animal Health (WOAH) and the Food and Agriculture Organization of the United Nations (FAO).

This paper presents a new AI dataset, in which EMPRES-i is enriched thanks to the genome sequence data of Avian Influenza cases affecting bird species from 2012 to 2021, publicly provided by the Bacterial and Viral Bioinformatics Resource Center (BV-BRC). This dataset is obtained by automatically linking sequence information in BV-BRC to the AI events in EMPRES-i, which results in “*putatively*” linked events between these two sources. The collected data is structured by nature, but it is preprocessed and normalized for the purpose of high-quality data linkage. Moreover, several data linkage strategies and missing information handling are introduced. To show the usefulness of our dataset, we quantitatively evaluate the proposed strategies in randomly sampled events and present in the end a diffusion network inference task.

Specifications Table

**Table:** 

Subject	Computer Science: Information System
Specific subject area	Linked Avian Influenza Epidemiological and Genomic Data
Type of data	Tabular data (*.csv). Raw and Standardized.
Data collection	The surveillance data were retrieved from EMPRES-i and the sequence information from BV-BRC. Disease: Avian Influenza, Host: Birds, Study period: 2012–2021.
Data source location	The data are hosted on the INRAE Dataverse in the context of the MOOD (MOnitoring Outbreaks for Disease surveillance in a data science context) project^1^.
Data accessibility	Repository name: Data INRAE (Dataverse) Data identification number: doi: 10.57745/JNA7N9 Direct URL to data: https://doi.org/10.57745/JNA7N9
Related research article	None.

## 1 Value of the Data

• This dataset contributes to the available resources in the field of Avian Influenza surveillance and epidemic intelligence.
• It completes the genetic information of the spatio-temporal AI events.
• It is useful for epidemiologists and computer scientists for studying AI transmission dynamics.
• It can be used for evaluation or training purposes for classification and network inference tasks.

## 2 Background

The emergence and spread of Avian Influenza (AI) has serious consequences for animal health and a substantial socio-economic impact for agriculture. For instance, the 2021–2022 season have witnessed the largest observed highly pathogenic avian influenza (HPAI) cases in Europe so far, with a total of 2467 outbreaks in poultry, 3573 HPAI events in wild birds, and 48 million birds culled in the affected establishments^2^. Due to this highly contagious nature, it is critical to monitor the ongoing AI cases. To this aim, epidemic intelligence has been used to remedy this animal health emergency.

For a global epidemic monitoring approach, several national and international surveillance systems are used, the well-known one being the EMPRES-i database from the World Organisation for Animal Health (WOAH) and the Food and Agriculture Organization of the United Nations (FAO) [1]. This database regularly collects structured and verified official animal health threats, hereafter referred to as epidemiological events (or events for short), through routine national surveillance systems and public health authorities. As a result, it is a well-populated official database for Avian Influenza and has been often used as reference gold standard in the literature [2], [3], [4].

Currently, EMPRES-i does not provide any linkage between its epidemiological events and the corresponding genome sequence information. However, combining epidemiological information and geomapping in the analysis of AI can contribute to a better understanding and description of AI epidemiology. In the literature, [5] has already proposed in 2013 to enhance the EMPRES-i database for H5N1 and H7N9 serotypes, but their genetic module is not operational anymore. For this reason, we propose in this paper a new AI dataset, in which EMPRES-i is enriched with the genome sequence data of AI cases, publicly provided by the Bacterial and Viral Bioinformatics Resource Center (BV-BRC) [6]. This new dataset concerns the AI events in EMPRES-i, affecting bird species from 2012 to 2021. It is worth highlighting that the AI host types (e.g. mammals) other than birds are not in scope of this work.

## 3 Data Description

Our goal in this work is to enrich the AI cases in the EMPRES-i database $D_{EMPRES-i}$ with genetic information provided by the BV-BRC database $D_{BV-BRC}$. As explained later in Section 3.2, we employ two linkage strategies to associate the genetic information in $D_{BV-BRC}$ to $D_{EMPRES-i}$: *1-to-1* and *1-to-many* linking. In the first one, a genome sequence can be associated to *only one* EMPRES-i event, whereas this unicity constraint is omitted in the second one in order that a genome sequence can be linked to *multiple* EMPRES-i events. The first (resp. second) strategy is more strict (resp. relaxed) and always produces less (resp. same or more) linked data compared to the other strategy. Ideally, the *1-to-1* strategy must be the only choice for such a task. However, due to possibly erroneous and imperfect information in our databases, it might be beneficial to use in practice the *1-to-many* strategy to have more linked cases between $D_{BV-BRC}$ and $D_{EMPRES-i}$, depending on the application at hand. Finally, the application of these two strategies results in two datasets, that we call $D_{strict}$ and $D_{relaxed}$, respectively. We detail their distributions per year and disease serotype in Table 1. In total, $D_{strict}$ and $D_{relaxed}$ contain 4797 and 13,300 events, respectively.

**Table 1:** Statistics on the two datasets $D_{strict}$ and $D_{relaxed}$. The columns represent all possible AI strains found in $D_{EMPRES-i}$, grouped by the H subtype for simplicity, and the rows correspond to yearly periods from 2012 to 2021. The last column (resp. row) summarizes the statistics by row (resp. column). Finally, each entry in the table has the form of x/y/z, in which x, y and z represent the number of events in $D_{strict}$, $D_{relaxed}$ and $D_{EMPRES-i}$, respectively.

Year	H3	H5	H6	H7	H9	H10	Total
**2012**	0/0/0	231/544/732	0/0/0	6/42/52	185/187/192	0/0/0	**422/773/976**
**2013**	0/0/0	164/474/688	0/0/0	273/347/430	172/172/180	1/1/1	**610/994/1,299**
**2014**	0/0/0	263/964/1,266	0/0/0	600/690/705	24/25/26	2/2/3	**889/1,681/2,000**
**2015**	0/0/0	992/2,662/3,062	0/0/0	87/303/312	16/16/17	0/0/0	**1,095/2,981/3,391**
**2016**	0/0/0	453/1,548/2,049	0/0/0	102/192/234	9/9/21	0/0/0	**564/1,749/2,304**
**2017**	0/0/0	561/2,360/3,449	0/0/1	266/1,035/1,040	4/4/6	0/0/0	**831/3,399/4,496**
**2018**	0/0/0	162/403/780	0/0/0	9/18/31	4/4/4	0/0/0	**175/425/815**
**2019**	7/7/7	29/86/239	0/0/0	1/8/62	1/1/1	0/0/0	**31/95/309**
**2020**	0/0/0	89/414/1,554	0/0/0	1/3/44	0/0/0	0/0/0	**90/417/1,598**
**2021**	0/0/0	89/785/3,723	0/0/0	1/1/7	0/0/0	0/0/0	**90/786/3,729**
**Total**	**7/7/7**	**3,033/10,240/17,542**	**0/0/1**	**1,346/2,639/2,917**	**415/418/447**	**3/3/4**	**4,797/13,300/20,918**

It is worth mentioning that both $D_{strict}$ and $D_{relaxed}$ are not the fusion of the AI cases from $D_{EMPRES-i}$ and $D_{BV-BRC}$, rather they are directly related to the EMPRES-i database. For this reason, they contain only the serotypes and avian host names available in $D_{EMPRES-i}$ (see the online supplementary material for all the available information). Although there are some discrepancies in the disease/host focus of both databases and they are therefore complementary (see the online supplementary material for more details and comparative results), merging them to obtain a single large AI database is not in scope of this work.

## 4 Experimental Design, Materials and Methods

This section details how we obtain our final datasets $D_{strict}$ and $D_{relaxed}$ by using data normalization (Section 3.1) and data linkage strategies (Section 3.2).

### 4.1 Data processing and normalization

**Fig. 1:**
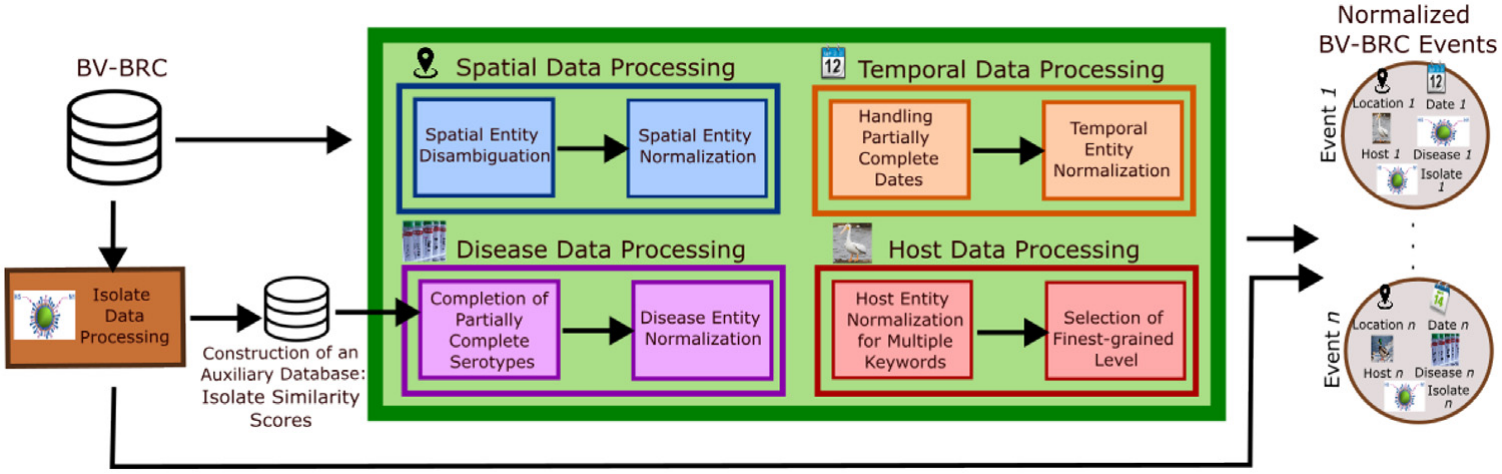
Workflow regarding our data processing and normalization tasks applied to the events of BV-BRC.

In this section, we describe the data processing and normalization tasks applied to $D_{BV-BRC}$, as illustrated in Fig. 1. These tasks are required for data linkage explained later in Section 3.2. Here, the data processing operations aim to clean and reformat the raw entries, and to complete the missing information, if possible. These are essential operations, because raw entries can be sometimes problematic, as illustrated with an example in Table 2.

**Table 2:** Example of raw texts associated with an isolate record in $D_{BV-BRC}$ regarding its spatial, temporal, disease and host information. These information can be sometimes problematic, as illustrated in this example. For the sake of completeness, we list here all possible issues with the raw texts. **1) Spatial:** A region information can be provided with an ISO or ADM1 code. For instance, in this example the code *ENG* correspond to England. Furthermore, when several spatial attributes are provided, they might not be ordered in a hierarchical manner. For instance, in this example *Skelmersdale* is a town in *England*. Therefore, it must be placed after *England* in order to respect the hierarchal order. **2) Temporal:** This information can be partially complete. For instance, in this example the day information is missing. **3) Disease:** This information can be partially complete. For instance, in this example the N subtype information is missing (e.g. the N1 part in H5N1). **4) Host:** This information can be very detailed (e.g. with gender and age) and can have multiple host keywords. For instance, in this example the keywords *mallard, duck* and *bird* are found in the same description.

Location	Date	Disease	Host	Isolate
United Kingdom, Skelmersdale, ENG	03–2021	H5	mallard duck; bird; gender Female; age Adult	{PB1: 11320.124852, PB2: 11320.124884}

For the normalization task, we use the same normalization operations used in [4], applied for $D_{EMPRES-i}$. This task consists in normalizing the attributes of each event by transforming a raw text into one of well-defined taxonomy classes (i.e. hierarchical representation), assuming that the events are defined as in Section 3.1.1. Concretely, these processing and normalizing operations concern the spatial (Section 3.1.2), temporal (Section 3.1.3), disease (Section 3.1.4) and host (Section 3.1.5) information of the events in $D_{BV-BRC}$.

#### 4.1.1 Event definition

We define an event throughout this work as the detection of the AI virus for a specific host at a specific date and in a specific location. Moreover, we also consider its genetic information, when available. For instance, this information is available for BV-BRC and our final datasets $D_{strict}$ and $D_{relaxed}$, but not for EMPRES-i. All these different information constitute the attributes of an event. Note that in an event, a location is expressed as the names of polygons (e.g. country or city names), but its spatial coordinates can be easily retrieved through a geocoding tool thanks to our normalization step (see Section 3.1.2). Moreover, the genetic information in $D_{BV-BRC}$ is organized as virus isolates. An isolate is the name for a virus that we have isolated from an infected host. In an AI isolate, there are in total eight segments: *PB1 (polymerase basic 1), PB2 (polymerase basic 2), PA (polymerase acidic), HA (hemagglutinin), NP (nucleoprotein), NS (nonstructural protein), NA (neuraminidase)* and *M (matrix protein)* [7]. However, in some isolates in $D_{BV-BRC}$, it is possible to have only some of these segments, which gives the partial view of an isolate. For instance, in the example of Table 2, only the segments PB1 and PB2, out of eight, are present. Note that we even make use of these partial isolates in data linkage strategies explained in Section 3.2.

For comparison purposes, the attributes of an event are usually normalized. This normalization step allows representing an event attribute in a hierarchical manner, thanks to well-defined hierarchical taxonomy classes. For instance, we obtain the normalized event illustrated in Table 3, after the event normalization task is applied to the raw entries in Table 2. Note that each event attribute can have a different hierarchical level. For the sake of compactness and simplicity, we show in this work only the information available at the finest-grained level for each event attribute. For instance, the compact view of the normalized event in Table 3 is illustrated in Table 4.

**Table 3:** Normalized event representation in a hierarchical manner for the raw texts of an isolate record illustrated in Table 2 after the event normalization task is applied (see Section 3.1 for more details). For the sake of simplicity, we represent the isolate information as an additional event attribute, although it is disease-related information.

Hierarchy level	Location	Date	Disease	Host	Isolate
1	Europe	2021	avian flu	aves (bird)	{PB1: 11320.124852, PB2: 11320.124884}
2	United Kingdom	03–2021	H7	neognathae	
3	England	week 13	H7N9	galloanserae	
4	Lancashire	31-03-2021		anseriformes	
5	West Lancashire			anatidae	
6	Skelmersdale			anatinae	
7				anas (duck)	
8				anas platyrhynchos (mallard duck)

**Table 4:** Compact view of the normalized event in Table 3. Each column corresponds to an event attribute. We show in these columns only the information available at the finest-grained level.

Location	Date	Disease	Host	Isolate
Skelmersdale	31-03-2021	H7N9 serotype	mallard duck	{PB1: 11320.124852, PB2: 11320.124884}

#### 4.1.2 Spatial information

Each AI case in $D_{BV-BRC}$ has the spatial information. But, this information can be at different spatial scale from one case to another (country, city, etc). Next, we describe our spatial entity disambiguation and normalization steps.

First, we need to perform spatial entity disambiguation. Indeed, due to the hierarchical nature of this information, some values can be ambiguous, because there is not any rule regarding the attribute order. For instance, the city information can randomly be preceded or succeeded by its region name (see Table 2 for an example). This makes the normalization task difficult. Therefore, we use three geocoding tools (ArcGIS^3^, Nominatim^4^, GeoNames^5^) to solve this attribute order issue. The goal is not to normalize spatial entities, rather identifying which part of the text corresponds to spatial entity attributes. For instance, after solving the attribute order issue in Table 2, we find out that *Skelmersdale* is a town, which is contained in England.

Then, we perform the normalization of spatial entities. This task consists in assigning geographic coordinates to spatial entities. In this work, we perform this task with the gazetteer GeoNames, as done in [4]. For a given query of spatial entity, GeoNames outputs a ranked list of most appropriate geographic coordinates associated with the input text. We simply take the first result, associated with the desired country name. For instance, if GeoNames proposes two results for *Skelmersdale* with two different country information (e.g. *United Kingdom* and *Sweden*), then we keep the result with *United Kingdom*, which is the desired country name according to Table 2.

#### 4.1.3 Temporal information

Each AI case in $D_{BV-BRC}$ has also the collected date information, which is in the form of *YYYY-MM-DD*. However, this information in several cases is partially complete, in that the day and/or month information is missing. We handle these incomplete dates with two strategies. If the temporal information only misses the day attribute, we simply consider it the first day of its month. Otherwise, when both the day and month attributes are missing, we duplicate the event 12 times, one for each month. The last operation aims to ease the data linkage process between $D_{BV-BRC}$ and $D_{EMPRES-i}$. Finally, we normalize the temporal expressions according to the TIMEX3 annotation standard.^6^

#### 4.1.4 Disease information

The serotype information of some AI cases in $D_{BV-BRC}$ are partially completed, for instance H5 or N1 instead of H5N1. To make the data at hand more available in data linkage, we estimate their exact serotype information, thanks to our auxiliary database $D_{GS}$ of isolate similarity scores, obtained from all pairs of events in $D_{BV-BRC}$. This $D_{GS}$ auxiliary database is more detailed later in Section 3.2. Concretely, for a given event with partial serotype information, we first take from $D_{GS}$ the isolate similarity scores between the isolate in question and most likely other isolates, then the isolate with highest similarity score determine its exact serotype. For instance, if the serotype is H5, then we select in $D_{GS}$ all isolates with the H5 subtype (e.g. H5N1, H5N2, etc.). We normalize these disease values with custom taxonomy classes in order to group the serotypes within the same H subtype (e.g. H5N1 and H5N2 are grouped for H5).

#### 4.1.5 Host information

The host information can be very detailed (e.g. with gender and age). For this reason, we select only avian names through the NCBI Taxonomy database. In the end, an AI case in $D_{BV-BRC}$ can have multiple host keywords extracted (see Table 2 for an example). Then, for each AI case, we normalize these host keywords against the NCBI Taxonomy database [8], using a manually composed table of species name synonyms. Then, we keep the host name, which is at finest-grained level. For instance, if the keywords *mallard duck* and *duck* are both present, we keep only *mallard duck*.

### 4.2 Data linkage

**Fig. 2:**
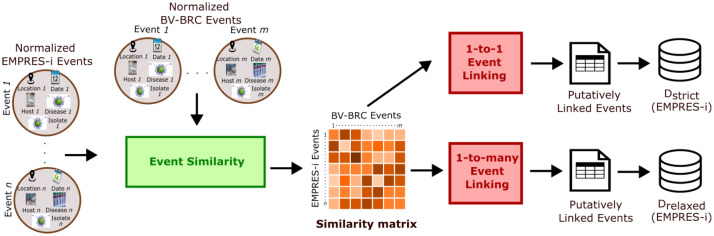
Workflow for event linking.

In this section, we take in input the preprocessed and normalized events (hereafter, simply *events*) from $D_{BV-BRC}$ and $D_{EMRPRES-i}$, as explained in Section 3.1. Our goal is to identify common (i.e. ”*putatively*” linked) events between $D_{BV-BRC}$ and $D_{EMRPRES-i}$ in an automatic manner, which is not a trivial task. We illustrate in Fig. 2 the workflow regarding this data linkage task. In the following, we first introduce how we compute the similarity of two events (Section 3.2.1), then pass to the data linkage strategies (Section 3.2.2).

#### 4.2.1 Event similarity

For event linking, we need to assess the similarity of two events in the presence of hierarchical data. This hierarchical data requires us to rely on an ontology-based semantic similarity measure. Due to this specificity, we use the similarity measure proposed in [4], which is similar to the state of the art measures, but tailored for epidemiological events. For the sake of clarity, we briefly explain it here.

In the similarity assessment, the main idea is that two events are considered similar, if 1) all their event attributes are identical or hierarchically linked, and 2) their event dates are close enough. Otherwise, they are penalized with a large negative value in the calculation in order that the underlying events are not linked. We slightly change the calculation proposed in [4] for the spatial attribute, tough. In its initial version, two events of different countries cannot be linked. We change it in order that two events of neighboring or geographically close countries to be linked. Concretely, we first calculate the similarity for each event attribute, and then we sum up the obtained values in order to get the final score. In the end, we obtain a similarity matrix, in which each entry correspond to the similarity score for a given event pair from $D_{EMRPRES-i}$ and $D_{BV-BRC}$ (see Fig. 2 for an illustration).

#### 4.2.2 Data linkage strategies

In this section, we present two linkage strategies: 1) 1-to-1 (Section 3.2.2.1) and 2) 1-to-many (Section 3.2.2.2) linking. In the first strategy, an event in $D_{BV-BRC}$ can be associated with only one event in $D_{EMRPRES-i}$, whereas in the second strategy the same event in $D_{BV-BRC}$ can be mapped to multiple events in $D_{EMRPRES-i}$. In both strategies, we rely on the similarity matrix S of $D_{BV-BRC}$ and $D_{EMRPRES-i}$. The term $S_{ij}$ represents the similarity score between events $e_{i}\in D_{BV-BRC}$ and $e_{j}\in D_{EMRPRES-i}$, and it is calculated as described in Section 3.2.1.

*1-to-1 Event Linking* As a first strategy, an event in $D_{BV-BRC}$ can be associated with only one event in $D_{EMRPRES-i}$. We propose to model this task as an assignment problem based on the matrix S, as already done in the literature (e.g. [9]). It can be solved through the well-known Hungarian algorithm [10]. In the end, we obtain a set of ”*putatively*” associated events between $D_{BV-BRC}$ and $D_{EMRPRES-i}$. Finally, in the solution of the assignment problem, some events might be assigned to other events with negative or weak positive similarity scores. Therefore, we perform a post-processing by removing the assignment results, whose similarity scores are lower than some threshold value. In the end, we obtain our final dataset $D_{strict}$.

*1-to-many Event Linking* As a second strategy, an event in $D_{BV-BRC}$ can be associated with multiple events in $D_{EMRPES-i}$ in order to cover as many events as possible in $D_{EMRPES-i}$. This means that each event in $D_{EMRPES-i}$ is associated to the event in $D_{BV-BRC}$ with highest similarity score. Concretely, for each event in $D_{EMRPES-i}$, we take the column-wise maximum in S. Finally, as in Section 3.2.2.1, some events might be assigned to other events with negative or weak positive similarity scores. Therefore, we only keep assignment results, whose similarity scores are lower than some threshold value. In the end, we obtain our final dataset $D_{relax}$.

## 5 Limitation: Missing Isolate Information

In our final datasets $D_{strict}$ and $D_{relaxed}$, some of their events has missing isolate information after the data linkage process, due to the different data sizes in $D_{BV-BRC}$ and $D_{EMPRES-i}$ and possibly erroneous and imperfect information in these sources. In this section, the goal is to show how we manage to handle the missing isolate information in $D_{strict}$ and $D_{relax}$, which can be beneficial for an application at hand (e.g. as our practical case in Section 6). In the following, we first introduce how we define isolate similarity (Section 4.1), then present the construction of an auxiliary database of isolate similarity scores (Section 4.2). Finally, we present our four strategies for the missing isolate information (Section 4.3).

### 5.1 Isolate similarity

Recall that in an AI isolate, there are in total eight segments: *PB1 (polymerase basic 1), PB2 (polymerase basic 2), PA (polymerase acidic), HA (hemagglutinin), NP (nucleoprotein), NS (nonstructural protein), NA (neuraminidase)* and *M (matrix protein)* [7]. Each segment is associated with a genome sequence. In the isolate similarity assessment, we compute the similarity value for each same segment pair (e.g. PB1 vs. PB1, PB2 vs. PB2), then take its average to obtain the final similarity score.

To compute the similarity of two segments (i.e. genome sequence), we rely on pairwise sequence alignment. This is the process of aligning two sequences to each other by optimizing the similarity score between them based on a predefined substitution matrix [11]. In this work, we use a default substitution matrix proposed in the Bio.Align Python package [12]. Finally, the obtained raw similarity score from the substitution matrix is normalized by the maximum similarity score obtained when each sequence is compared to itself.

### 5.2 Construction of an auxiliary database: isolate similarity scores

We take advantage of the large size of genome sequence information provided by BV-BRC to constitute an auxiliary database $D_{GS}$ of isolate similarity scores. These scores are obtained with the similarity measure explained in Section 4.1 for all pairs of temporally close events in $D_{BV-BRC}$ with complete serotype information (e.g. H5N8 vs. H5N8, H5N8 vs. H5N1). The temporal distance is fixed in such a way that two events of the same year or subsequent years are only kept (e.g. 2017 vs. 2017, 2017 vs. 2018, 2018 vs. 2017). Note that this auxiliary database is used for two purposes: 1) completion of partial serotype information (Section 3.1.4) and 2) handling missing isolate information in the datasets $D_{strict}$ and $D_{relax}$ (Section 4.3).

As an example, we show in Table 5 an excerpt from these scores for only the events with H5N8 serotype, occurring in South Africa and Namibia. In this table, we call *source* and *target* events to distinguish two events in the similarity calculation. Moreover, to show how many similarity scores are computed by serotype pair in $D_{GS}$, we show some statistics in Table 6. The very large numbers in this table highlight the importance of $D_{GS}$ and its capability of precise estimations in any task.

**Table 5:** Excerpt from $D_{GS}$. Only some events with H5N8-H5N8 serotype pair, occurring in South Africa and Namibia, are shown. In this table, we call *source* and *target* events to distinguish two events in the similarity calculation.

Source country vs. Target country	Source sequence name	Source year	Target sequence name	Target year	Similarity score
			*A/African penguin/ South Africa/18010422/2018*	2018	0.99
South Africa vs South Africa	*A/African oystercatcher/ South Africa/18030214/2018*	2018	*A/Guinea fowl/ South Africa/17080243/2017*	2017	0.92
			*A/African penguin/ South Africa/476266/2018*	2018	0.92
			*A/African penguin/ Namibia/ 146S/2019*	2019	0.71
South Africa vs Namibia	*A/African oystercatcher/ South Africa/18030214/2018*	2018	*A/African penguin/ Namibia/ 218-1/2019*	2018	0.61
			*A/African penguin/ Namibia/ 288-1/2019*	2018	0.80

**Table 6:** Sizes of some serotype pairs in $D_{GS}$.

Serotype pair	Size
H5N1 vs H5N1	1,308,268
H5N8 vs H5N8	605,345
H7N9 vs H7N9	545,614
H9N2 vs H9N2	34,845,712
H5N1 vs H5N8	567,071
H5N8 vs H7N9	356,169

### 5.3 Handling missing isolate information

If two events in $D_{EMRPES-i}$ have their associated isolate information, we can simply compute the isolate similarity between them. However, despite of two linkage strategies proposed in Section 3.2.2, it is possible not to assign an isolate to an event in $D_{EMRPES-i}$. This can be due to automatic normalization issues or incompleteness of $D_{BV-BRC}$. To overcome this issue, we propose four strategies for handling the absence of isolation information in $D_{strict}$ and $D_{relax}$. The goal here is to take advantage of $D_{GS}$ in order to compute average similarity scores with respect to some selected event attributes.

Given event pairs, the first and second strategies are used when only one event has missing isolate information, and not the other one. In this case, the one with isolate information is referred to as *source* event, and the other as *target* event. The first strategy is used, when the sequence information of the source event, i.e. *source sequence*, and the country information of the target event, i.e. *target country*, are known in $D_{GS}$, given a serotype pair. We illustrate this with an example in Table 7. For instance, the first row corresponds to the average similarity score between *A/African oystercatcher/South Africa/18030214/2018*^7^ and *South Africa*. This similarity score is obtained by taking the average of all similarity scores obtained from event pairs, both occurring in South Africa with known isolate information. Otherwise, if the first strategy cannot be used, we use the second strategy, in that only source sequence is used to compute an average similarity score without taking the target country into account, as illustrated in Table 8.

**Table 7:** Illustration of the first strategy with H5N8-H5N8 serotype pair, which is used when only one event has missing isolate information.

Source sequence name	Target country	Similarity score
*A/African oystercatcher/South Africa/18030214/2018*	South Africa	0.95
	Zimbabwe	0.92
	Belgium	0.53
*A/African penguin/Namibia/146S/2019*	Namibia	0.99
	Nigeria	0.79
	Pakistan	0.64

**Table 8:** Illustration of the second strategy with H5N8-H5N8 serotype pair, which is used when only one event has missing isolate information.

Source sequence name	Similarity score
*A/African oystercatcher/South Africa/18030214/2018*	0.85
*A/African penguin/Namibia/146S/2019*	0.72

The third and fourth strategies are used when none of the two events has an isolate information. The third strategy relies on the country information of both events. If a pair of county names, for a given serotype, is known in $D_{GS}$, then we compute the average similarity score by taking all similarity scores obtained for event pairs with known isolate information, occurring in both countries. This is illustrated in Table 9. Otherwise, we use the fourth strategy. In this case, we compute the average similarity score by taking all similarity scores obtained from each pair of events with complete isolate information, without taking the country information into account. This is illustrated in Table 10.

**Table 9:** Illustration of the third strategy with H5N8-H5N8 serotype pair, which is used when none of the two events has an isolate information.

Country pair	Similarity score
South Africa vs South Africa	0.97
South Africa vs Zimbabwe	0.96
South Africa vs Belgium	0.56
Namibia vs Namibia	0.99
Namibia vs Nigeria	0.79
Namibia vs Pakistan	0.68

**Table 10:** Illustration of the fourth strategy with H5N8-H5N8 serotype pair, which is used when none of the two events has an isolate information.

Serotype pair	Similarity score
H5N5 vs H5N5	0.99
H5N8 vs H5N8	0.85
H5N5 vs H5N8	0.70

## 6 Quantitative Evaluation

In this section, we evaluate the proposed strategies to deal with the completion of partial serotype information (Section 3.1.4), data linkage between two event databases (Section 3.2.2) and handling missing isolate information in $D_{strict}$ and $D_{relax}$ (Section 4.3). For these assessments, we create a subset $D_{gt}$ of events from our data, which contains in total 500 randomly sampled events with complete isolate information. Since all the events in $D_{gt}$ have complete isolate information, we use the dataset $D_{gt}$ in our assessments as the ground-truth. Next, we detail our three quantitative evaluation tests by using $D_{gt}$ and show their corresponding results.

First, we evaluate how successful our proposed completion strategy in Section 3.1.4 is for dealing with partial serotype information. For this assessment, we create another dataset $D_{eval}$ of events by duplicating $D_{gt}$ and making the disease serotype information of all its 500 events partially complete (e.g. *H5* or *N1* instead of *H5N1*). Then, we perform the completion of partial serotype information based on the auxiliary database $D_{GS}$, as explained in Section 3.1.4, in order to compare the results with $D_{gt}$. As a result, our evaluation test finds out that the proposed strategy correctly estimate the complete serotype information in 444 events (0.89 in proportion).

Second, we are also interested in the evaluation of the event linking process between two event databases, as explained in Section 3.2.2. For this assessment, we first create multiple datasets of events by duplicating $D_{gt}$ and perturbing the events to the extent of the perturbation parameter $p_{pert}$, which is in the range of [0,1]. Concretely, the perturbation process first randomly selects with the probability of $p_{pert}$ the attributes of an event for which the modification is done, and it then makes the selected attributes coarser (i.e less precise) based on the corresponding taxonomy trees. When the value of $p_{pert}$ is close to 0 (resp. 1), this means that the events of $D_{gt}$ are modified to small (resp. large) extent and they are very (resp. not very) close to their initial counterpart. In our evaluation test, we use four $p_{pert}$ values, which are 0.25, 0.50, 0.75 and 1.00, and this results in four datasets of events, which we call $D_{p_{pert}=0.25}$, $D_{p_{pert}=0.50}$, $D_{p_{pert}=0.75}$ and $D_{p_{pert}=1.00}$, respectively.

Then, we apply the *1-to-1* and *1-to-many* event linking strategies between $D_{gt}$ and all four perturbed datasets of events. Ideally, the linking process is supposed to link the same events, which can be verified based on their event identifiers. If the process finds (resp. does not find) the same events, we say that they are correctly (resp. falsely) linked. It is also possible that the linking process fails to link some event pairs in two event datasets (i.e. unlinked cases). We show in Table 11 the proportion of the correctly and falsely linked event pairs, as well as that of unlinked cases, for four perturbed event datasets. We see from the table that the performance of event linking gets worse when the perturbation degree increases, as expected. However, the proportion of correctly linked cases is still large enough (i.e. the scores of 0.75 and 0.81), even when $p_{pert}=1.00$.

**Table 11:** Evaluation of the event linking process between $D_{gt}$ and four perturbed datasets of events $D_{p_{pert}=0.25}$, $D_{p_{pert}=0.50}$, $D_{p_{pert}=0.75}$ and $D_{p_{pert}=1.00}$.

Strategy	Description	Evaluation with $p_{pert}=0.25$	Evaluation with $p_{pert}=0.50$	Evaluation with $p_{pert}=0.75$	Evaluation with $p_{pert}=1.00$
1-to-1	Proportion of correctly linked cases	0.95	0.92	0.85	0.75
	Proportion of falsely linked cases	0.03	0.05	0.08	0.14
	Proportion of unlinked cases	0.02	0.03	0.07	0.11
1-to-many	Proportion of correctly linked cases	0.98	0.94	0.88	0.81
	Proportion of falsely linked cases	0.01	0.02	0.05	0.07
	Proportion of unlinked cases	0.01	0.03	0.07	0.12

Finally, we also assess how correct the estimation of the applied four strategies is for handling missing isolate information in $D_{strict}$ and $D_{relax}$ (Section 4.3). Recall that these strategies are applied when at least one event has missing isolate information for the isolate similarity calculation of two events. We perform our evaluation test in two parts. In the first part, we explore to what extent the proposed four strategies are in practice used. To do so, we create another dataset $D_{eval}$ of events by duplicating $D_{gt}$ and removing the isolate information of its 250 events. We show in Table 12 the proportion of use of these four strategies in the pairs of events in $D_{eval}$, when when at least one event has missing isolate information. We see from this table that the strategies 1 (with the score of 0.54) and 3 (with the score of 0.30) are prevalently used in practice.

**Table 12:** Proportions of use of four strategies proposed in Section 4.3 for the pairs of events in $D_{eval}$, when when at least one event has missing isolate information (see the column *Isolate information*).

Strategy	Isolate information	Proportion of use
1 (source sequence vs. target country)	only one of the events	0.54
2 (source sequence only)	only one of the events	0.05
3 (source country vs. target country)	none of events	0.30
4 (default, serotype pair)	none of events	0.11

**Fig. 3:**
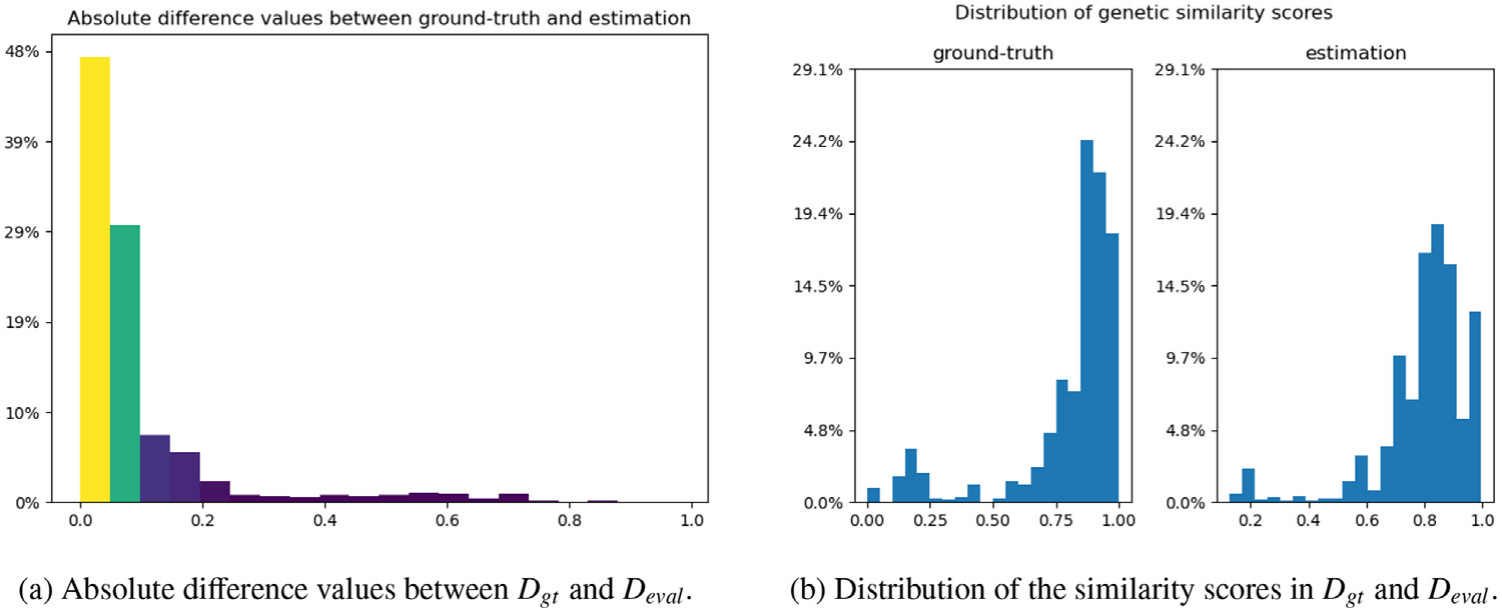
Evaluation of how close the computed isolate similarity scores in $D_{eval}$ with the four strategies proposed in Section 4.3, compared to $D_{gt}$.

In the second part, we also rely on $D_{gt}$ and $D_{eval}$, and assess how close the computed isolate similarity scores in $D_{eval}$ are after the estimation with the four strategies, compared to $D_{gt}$. For this assessment, we first separately compute the isolate similarity among pairs of events in $D_{gt}$ and $D_{eval}$. Then, we calculate the absolute difference values for the same event pairs in $D_{gt}$ and $D_{eval}$ to see how close these results are. Note that the same event pairs in $D_{gt}$ and $D_{eval}$ can be verified based on their event identifiers. We show in Fig. 3a the absolute difference values between $D_{gt}$ and $D_{eval}$. We see that approximately 80 % of the estimated scores are in the error range of [0,0.1] (i.e. yellow and green bars). Furthermore, for the sake of completeness, we also show in Fig. 3b the distribution of the calculated similarity scores in $D_{gt}$ and $D_{eval}$, before the calculation of absolute difference values. We observe that their overall distributions are sufficiently similar, with some small skewness differences.

## 7 Practical Case

To illustrate the usefulness of our datasets of AI events, we present a diffusion network inference task at meta-population level, publicly available online^8^. A network inference problem consists in estimating the underlying network structure, i.e. complete information on edge connectivity, node existence and the exact edge weights, from the event data at hand. In our context, the nodes and edges in the network to be inferred correspond to the spatial zones at ADM1 level (i.e. first level of subnational boundaries) and the disease transmissions among them, respectively. Generally speaking, we only know when an AI event occurs, but not exactly from where it is propagated, i.e. the underlying transmission dynamics among the zones. Hence, this network inference task aims to unveil the hidden AI transmission information in the presence of the temporal, spatial and genetic information of AI events.

To perform this task we adapt the method proposed in [13] to our dataset $D_{relax}$^9^ Briefly, [13] adopts a space-time diffusion model and a survival analysis framework for estimating the network structure. We simply extend their work by including the genetic information of AI events. Similar to [13], we use Rayleigh distribution with the parameter α to model the temporal distances among the events, and Exponential distribution with the parameters β and γ for the spatial and genetic distances, respectively. The values of α are what we estimate from the network inference problem and the values of the parameters β and γ are fixed to 0.01.

In this practical case, for illustrative purposes we select only a subset of our dataset, corresponding to the AI H5N8 events occurred between October and December 2016 in Europe. This period corresponds to the beginning of the H5N8 wave, which is the largest in the EU in terms of number of poultry outbreaks, geographical extent and number of dead wild birds. [14]. There are in total 606 events, in which 75 events do not have any isolate information despite of our data linkage strategy. We rely on the four strategies of handling mission isolate information, as explained in Section 4, in order to compute the isolate similarity values among these 75 events and the rest. To show the interest of including additional information, we sequentially infer three networks $G_{t}$, $G_{st}$ and $G_{stg}$ for time-only, space-time and space-time-genetic information, respectively. We filter out the edges, whose weight is lower than 0.05 to keep only the pertinent ones. We evaluate the obtained results in a qualitative manner based on the phylogenetic analysis conducted by [15], which estimates the transmission flows among AI H5 cases in Eurasia for the period 2016-17. In [15], the authors mainly find out that the virus is carried by wild birds during autumn migration 2016 to wintering locations in Europe through two main flows: 1) Russia → countries around Baltic Sea → Netherlands → France and 2) Russia → Ukraine → Hungary.

**Fig. 4:**
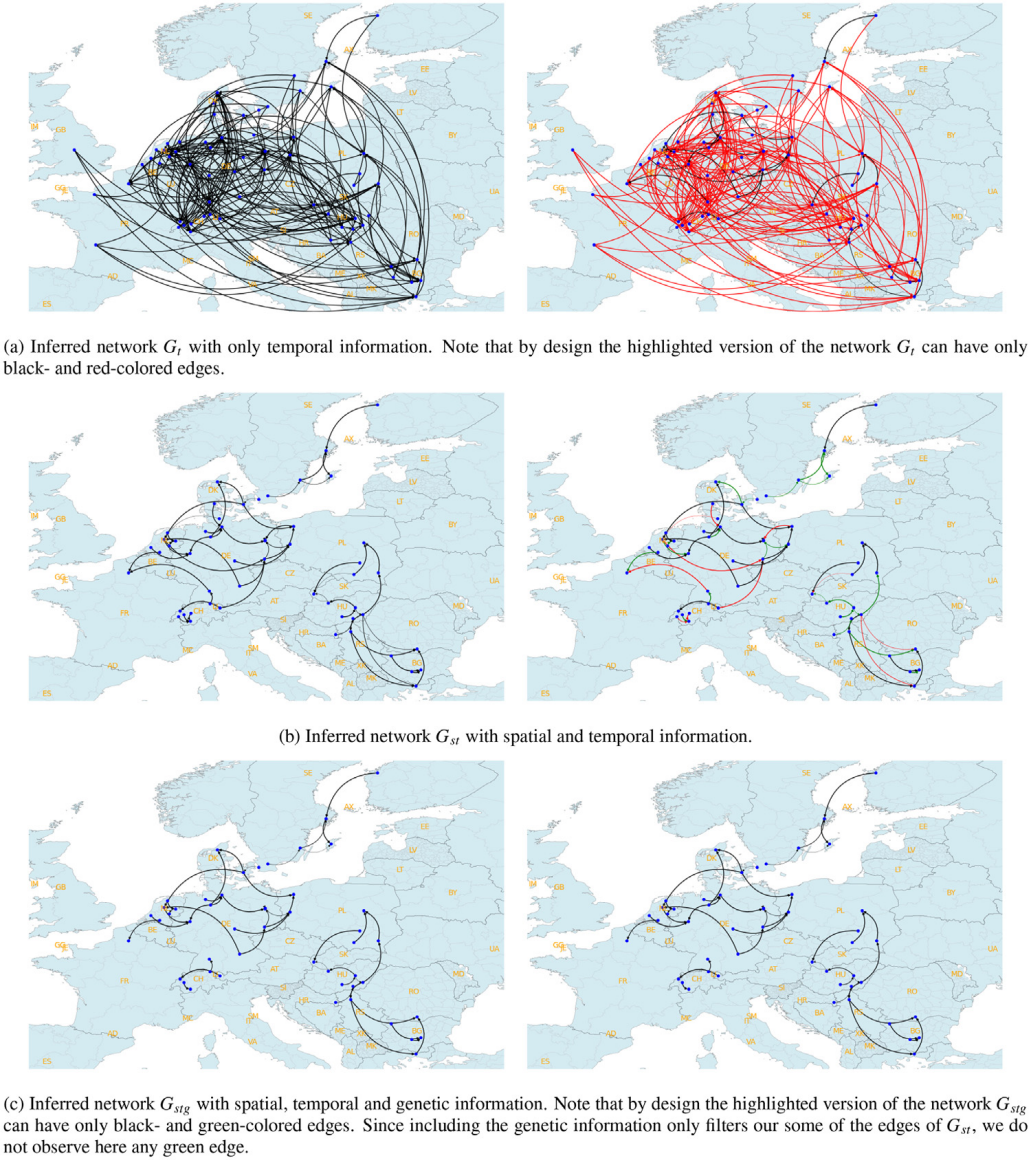
Our three inferred networks $G_{t}$, $G_{st}$ and $G_{stg}$ based on the AI H5N8 events in Europe for the period October-Decembre 2016. In (a), (b) and (c), a network is plotted twice for the sake of clarity. The first one corresponds to the obtained network and the second one represents the highlighted version of the first one. These highlights are based on the network pairs $G_{t}\;-\;G_{st}$ and $G_{st}\;-\;G_{stg}$, and show the evolution of the edges from $G_{t}$ to $G_{st}$ and from $G_{st}$ to $G_{stg}$, respectively. Finally, country codes in ISO 3166-1 alpha-2 standard are shown in all maps.

We first visualise the inferred networks $G_{t}$, $G_{st}$ and $G_{stg}$ in Fig. 4. In this figure, a network is plotted twice for the sake of clarity. The first one corresponds to the obtained network and the second one represents the highlighted version of the first one. These highlights are based on the network pairs $G_{t}\;-\;G_{st}$ and $G_{st}\;-\;G_{stg}$, and show the evolution of the edges from $G_{t}$ to $G_{st}$ and from $G_{st}$ to $G_{stg}$, respectively. An edge is colored in black, if it exists in both networks. Otherwise, its color is red in $G_{t}$ (resp. $G_{st}$), if it appears only in $G_{t}$ (resp. $G_{st}$) and not in $G_{st}$ (resp. $G_{stg}$). These red edges $G_{st}$ (resp. $G_{stg}$) indicate that they are filtered out from $G_{t}$ (resp. $G_{st}$). Similarly, an edge is colored in green in $G_{st}$ (resp. $G_{stg}$), if it appears only in $G_{st}$ (resp. $G_{stg}$) and not in $G_{t}$ (resp. $G_{st}$). These green edges in $G_{st}$ (resp. $G_{stg}$) indicate that they are inferred thanks to the inclusion of the spatial and (resp. spatio-genetic) information.

We can summarize the results in three points. First, we see from Fig. 4 that the network inference task with additional spatial and genetic information make the networks sparser (i.e. $G_{st}$ compared to $G_{t}$ and $G_{stg}$ compared to $G_{st}$), as indicated with the existence of multiple red edges in $G_{t}$ for $G_{st}$ and $G_{st}$ for $G_{stg}$. This can be seen as a filtering step towards reaching most likely transmission pathways. For instance, including the genetic information allows keeping only the transmission from Netherlands to the North of France by filtering out the transmission from Germany. Second, adding the genetic information does not add any new (i.e. green) edges in $G_{stg}$. Moreover, we only observe some slight differences between $G_{st}$ and $G_{stg}$. This indicates that the spatial and genetic distances between events are mostly correlated. Finally, our findings are mainly in line with the results in [15]. Overall, the benefit of including the genetic information is visually shown.

## Ethics Statement

No conflict of interest exists in this submission. The authors declare that the work described in this paper is original and not under consideration for publication elsewhere, in whole or in part. Its publication is approved by all the authors listed.

## CRediT authorship contribution statement

**Nejat Arınık:** Methodology, Software, Data curation, Writing – review & editing. **Roberto Interdonato:** Writing – review & editing. **Mathieu Roche:** Writing – review & editing. **Maguelonne Teisseire:** Writing – review & editing.

## Data Availability

DataverseLinked Avian Influenza Epidemiological and Genomic Data for Epidemic Intelligence (2012-2021) (Original data).

## Footnotes

1 https://mood-h2020.eu/.

## Footnotes

2 http://www.ecdc.europa.eu/en/news-events/2021-2022-data-show-largest-avian-flu-epidemic-europe-ever.

3 https://www.arcgis.com.

4 https://nominatim.org.

5 https://www.geonames.org.

6 http://timeml.org/site/publications/timeMLdocs/timeml_1.2.1.html.

7 We use the same textual format as in the database $D_{BV-BRC}$.

8 https://github.com/arinik9/AIAGIS.

9 We could also use the dataset $D_{strict}$.
